# THE MULTIDIMENSIONAL SUCCESSFUL AGING SCALE: A LONGITUDINAL VALIDATION STUDY

**DOI:** 10.1093/geroni/igae098.3541

**Published:** 2024-12-31

**Authors:** Edwin Ka Hung Chung, Dannii Y Yeung

**Affiliations:** City University of Hong Kong, Hong Kong, China (People’s Republic); City University of Hong Kong, Hong Kong, China (People’s Republic)

## Abstract

The diversified measurement tools of successful aging have restricted comparison of findings between studies and hindering development of this field advancement (Cosco et al., 2014). This study thereby aimed to validate the 25-item Multidimensional Successful Aging Scale (MSAS) through examining its overall structure, measurement invariance, and predictive validity. Four hundred and fourteen community-dwelling older Chinese adults (Mage=68.92 years, SD=6.64, range=60–91; 59.9% females) were assessed on the MSAS in conjunction with demographic, physical and psychological measures at baseline (Time 1) and at 6 months (Time 2). The results of confirmatory factor analysis first revealed a good model fit of the 9-factor model of the MSAS in the two assessments, and indicated that the 9-factor model fitted the data better than other higher-order factor models. In addition to the metric invariance across age and gender groups as well as strict invariance across groups of individuals with and without chronic illness, a strict form of longitudinal invariance was shown at the 6-month interval. Furthermore, the overall results of hierarchical linear regression analyses indicated that, among all dimensions of MSAS in Time 1, positive attitudes and perceived constraints were more predictive of depressive symptoms, functional ability, and pain in Time 2. Overall, the present study advances the previously established psychometric properties of MSAS (Chung & Yeung, 2021; 2022) by demonstrating its temporal stability and predictive value. Findings also support the multidimensional nature of successful aging, and provide health care practitioners and policymakers a more holistic understanding of what is important to older adults.

